# A Case Report of Cardiogenic Syncope Due to Loperamide Abuse: Acute Presentation and Novel Use of Buprenorphine

**DOI:** 10.5811/cpcem.2021.3.51152

**Published:** 2021-05-10

**Authors:** David J. Betting, James A. Chenoweth, Angela F. Jarman

**Affiliations:** UC Davis Medical Center, Department of Emergency Medicine, Sacramento, California

**Keywords:** Loperamide, buprenorphine, dysrhythmia, syncope

## Abstract

**Introduction:**

Loperamide is a non-prescription anti-diarrheal agent targeting μ-opioid receptors in the intestinal tract. At high doses it crosses the blood-brain barrier, where μ-opioid agonism can cause euphoric effects. Misuse has been increasing for both the euphoric effects and as an alternative treatment for opioid dependence and withdrawal.

**Case Report:**

Here we report the case of a 30-year-old woman presenting with syncope, who was found to have severe myocardial conduction delays in the setting of chronic loperamide abuse.

**Conclusion:**

Treatment with sodium bicarbonate and hypertonic sodium resulted in improvement of her conduction abnormalities. Prior to discharge she was initiated on buprenorphine for her opioid use disorder.

## INTRODUCTION

Loperamide is an over-the-counter synthetic opioid used to treat diarrhea. According to the National Poison Data System there has been a 91% increase in the reported nonmedical use of loperamide between 2010–2015.^1^ At therapeutic dosing, the drug’s effects are mainly targeted to the μ-opioid receptors in the intestinal mucosa. However, due to its relatively inexpensive cost and ease of accessibility there are reports of patients taking up to 800 milligrams (mg) per day for its euphoric effects. Additionally, high-dose loperamide is being used by some with opioid dependence as an over-the-counter treatment for opioid withdrawal symptoms.

Multidrug P-glycoprotein efflux pumps found on the digestive tract and blood-brain barrier (BBB) are normally effective at keeping the drug isolated to the digestive system and out of the brain at therapeutic drug concentrations. At higher doses, however, loperamide can overcome these pumps allowing it to enter the systemic circulation and cross the BBB resulting in stimulation of central nervous system opioid receptors. This can manifest as euphoria and result in significant respiratory depression.^2^,^3^ Additionally, there are reports of patients co-administering medications that inhibit the P-glycoprotein pumps (quinidine or piperine, found in black pepper) or slow down metabolism of the loperamide using cytochrome P450 3A4 inhibitors (cimetidine, grapefruit juice) to prolong its half-life.^2^

In June 2016 the US Food and Drug Administration released a warning regarding loperamide abuse and life-threatening adverse cardiac events.^4^ There have been multiple reports of QRS and QTc prolongation, torsades de pointes (TdP), Takotsubo cardiomyopathy, and cardiac arrest associated with loperamide abuse.^2^,^5^,^6^ Despite these warnings there has been a continuing trend toward abuse of loperamide with some touting it as the “poor man’s methadone.” Here we describe a case of cardiac toxicity and the novel subsequent treatment of loperamide abuse with medication for opioid use disorder (MOUD) using buprenorphine/naloxone.

## CASE REPORT

A 30-year-old female was brought in by ambulance after a witnessed syncopal event while shopping. Emergency medical services were called by bystanders, and upon initial evaluation a 12-lead electrocardiogram (ECG) revealed a wide complex tachycardia. The patient initially refused transport despite reporting another syncopal event earlier that day, stating that these episodes were common for her. Eventually, after speaking with medical control at our facility, the patient agreed to come to the hospital for further evaluation. On arrival to the emergency department (ED) the patient was alert and oriented with a heart rate of 77 beats per minute, blood pressure of 135/93 millimeters mercury, respiratory rate of 20 breaths per minute, oxygen saturation of 100% on room air, and a temperature of 36.6°C.

**Image 1:**
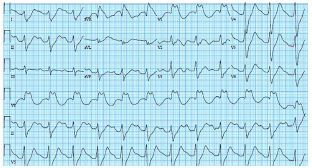
Initial emergency department electrocardiogram, prior to treatment initiation of the patient’s loperamide ingestion. The patient had a normal heart rate, but widened QRS and QTc with a terminal R wave in aVR.

She had no remarkable physical exam findings except for a small forehead abrasion suffered during her recent syncopal event. During the review of her social history the patient reported that she had been taking 50–200 (2 mg) tablets of loperamide every day for a year and a half. Over the prior six months she had been having more frequent syncopal episodes that were preceded by a feeling of heart fluttering. She did not seek care because she had no other symptoms and returned to her baseline immediately after the episodes. She denied prior opioid use but did have a prior history of alcohol abuse. She had been incarcerated a year earlier, during which time she had learned about and started using loperamide for its euphoric effects. She had also recently been briefly hospitalized for bradycardia, hypotension, nausea, vomiting, and diarrhea, possibly from unrecognized loperamide withdrawal. Her initial ECG, performed on arrival in the ED, showed a rate of 77 beats per minute, a tall R wave in aVR, QRS of 194 milliseconds (ms) (reference range: 80–100), and a QTc of 777 ms (350–460) (Image 1).

**Image 2:**
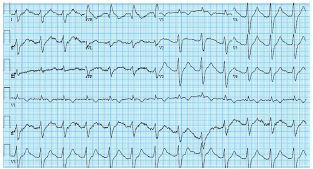
Electrocardiogram following treatment with intravenous magnesium, calcium chloride, five boluses of sodium bicarbonate, and a 30 ml bolus of 3% hypertonic saline in the emergency department. Her QRS and QTc remained prolonged and the terminal R wave in aVR is still present.

As treatment for her widened QRS and prolonged QTc interval the patient was given 2 grams (g) of magnesium, 1 g of calcium chloride, and five 50 milliequivalents of sodium bicarbonate boluses, given as intravenous pushes approximately five minutes apart for five doses. She then received a 30-milliliter bolus of 3% hypertonic saline due to concerns that further alkalinization could lower her potassium and potentially worsen QTc prolongation (serum electrolyte values were not yet available). Despite these therapies, an ECG performed just prior to admission to the medical intensive care unit (ICU) still showed a QRS of 180 ms and a QTc of 717 ms (Image 2). Her initial electrolyte values on presentation to the ED were as follows: potassium 4.4 millimoles per liter (mmol/L) (reference range: 3.3–5.0 mmol/L); magnesium 1.8 mg/deciliter (dL) (1.5–2.6 mg/dL); and total calcium 9.0 mg/dL (8.6–10.5 mg/dL).

**CPC-EM Capsule**

What do we already know about this clinical entity?

*Loperamide is a non-prescription anti-diarrheal medication that acts by targeting gastrointestinal μ-opioid receptors to slow gastrointestinal motility.*

What makes this presentation of disease reportable?

*High doses of loperamide can exert systemic opioid effects and cause life-threatening cardiac conduction abnormalities.*

What is the major learning point?

*Identification and treatment of drug-induced cardiac conduction abnormalities, and subsequent opioid use disorder management.*

How might this improve emergency medicine practice?

*Learning to rapidly identify and treat drug-related conduction abnormalities can be lifesaving and treating the opioid use disorder may help prevent reoccurrences.*

**Image 3:**
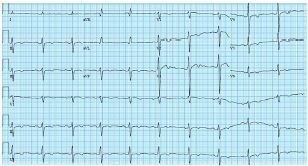
In the intensive care unit the patient was started on a sodium bicarbonate drip and frequent electrolyte and pH evaluations. Electrocardiogram prior to patient discharge showed improvement in the QRS and QTc, with near resolution of the terminal R wave in aVR.

The patient was then placed on an isotonic sodium bicarbonate drip for a day in the ICU with frequent ECG, pH, and electrolyte evaluations. After four days her QRS improved to 142 ms, QTc to 494 ms, and the R wave in aVR was greatly improved (Image 3).

Following this clinical improvement, the patient was started on MOUD with buprenorphine 4 mg daily. The day after discharge she returned to the hospital with nausea and vomiting concerning for ongoing withdrawal symptoms. She was found to again have a prolonged QRS of 134 ms and a QTc of 620 ms. Electrolytes were found to be in the normal ranges. Her QRS and QTc improved to 126 ms and 488 ms, respectively, with additional sodium bicarbonate treatment and maintenance of her serum electrolytes. Her nausea and vomiting also improved during her stay, and her buprenorphine was increased to 8 mg daily prior to discharge. On an outpatient visit three days later she had been continuing her buprenorphine with improvement of her withdrawal symptoms and denied any further cravings for loperamide.

## DISCUSSION

Here we present a case of a previously healthy young woman with increasing syncopal events preceded by palpitations in the setting of loperamide abuse. It has been shown previously that high doses of loperamide can result in significant toxicity. At high levels, loperamide can overwhelm the P-glycoprotein efflux pumps and cross the BBB resulting in euphoria and respiratory depression, similar to other opioid agonists. Like other opioids, the respiratory depression and mental status changes are dose dependent and appear to respond to naloxone treatment.^7^ However, unlike most opioids, at high doses loperamide causes antagonism of fast sodium channels and potassium-rectifying channels on cardiac myocytes causing widening of the QRS complex and prolongation of the QTc interval.^2^

These conduction abnormalities potentiate the risk for life-threatening arrhythmias such as torsades de pointes (TdP). Of note, conduction delays may pose an even greater risk to women, who have longer QTc intervals at baseline; this slower repolarization is thought to be caused by the relative lack of rectifier potassium channels in comparison to men.^8^ The risk of developing TdP is mitigated by tachycardia, which shortens the QTc interval and the risk of an “R on T” phenomenon; thus, this patient’s relatively slow rate combined with significant conduction delay put her at high risk of malignant arrhythmia. Given her presentation, it is likely that the palpitations she described prior to her syncopal events were due to a cardiac arrhythmia.

Prior reports have proposed treatment strategies for the cardiac conduction abnormalities attributed to high levels of loperamide and its metabolites. These include electrolyte repletion (potassium, magnesium, and calcium) to minimize QTc prolongation. Sodium bicarbonate or hypertonic saline can be used to increase the extracellular sodium concentration in an attempt to overcome the sodium channel blockade.^9^,^10^ Caution must be taken with sodium bicarbonate, however, as alkalinization may lead to decreases in serum potassium and ionized calcium, which could cause further QTc prolongation and other dysrhythmias. Class IB antiarrhythmic drugs, such as lidocaine, can also be used to reduce sodium channel blockade due to their rapid binding and dissociation times compared with other drugs.^11^ Finally, measures such as lipid emulsion rescue therapy, chemical or electrical pacing, and even extracorporeal membrane oxygenation have been attempted with varying degrees of success.^2^,^12^

Although not always thought of as a drug of abuse, loperamide is an opioid agonist and as such carries the risk of physiologic dependence and withdrawal, especially at the high doses seen in our patient. The use of buprenorphine for the treatment of opioid use disorder is well documented, but there is limited data on its use for loperamide.^13^,^14^ Our patient agreed to start MOUD with buprenorphine/naloxone to help treat her loperamide use disorder, and at her last documented office visit she was continuing treatment with no further cravings for loperamide.

## CONCLUSION

The prevalence of loperamide abuse has been increasing. and early recognition and treatment of the resulting cardiac toxicity by emergency physicians can be lifesaving. Syncopal events may be the first indication that a significant cardiac abnormality is present, and cardiac toxicity should be considered when reviewing ECGs on these patients. Patients with widened QRS or prolongation of the QTc interval, in particular, should be questioned about their medication and drug use including prescription, recreational, and over the counter. Emergency providers must be familiar with the treatment strategies for life-threatening cardiac conduction delays caused by sodium channel blockade. Addressing the underlying loperamide abuse should also be a priority as many people generally consider over-the-counter medications to be safe. This case also reinforces the potential efficacy and safety of buprenorphine in the treatment of non-traditional opioid use disorders.^13^–^15^
